# A Review of Combined Training Studies in Older Adults According to a New Categorization of Conventional Interventions

**DOI:** 10.3389/fnagi.2021.808539

**Published:** 2022-02-01

**Authors:** Marta Maria Torre, Jean-Jacques Temprado

**Affiliations:** Institut des Sciences du Mouvement, ISM UMR 7287, CNRS, Aix Marseille Université, Marseille, France

**Keywords:** aging, exercise, cognition, physical activity, combined training

## Abstract

Physical and cognitive training are effective to attenuate age-related declines of brain and cognition. Accordingly, interest in interventions that combine physical, motor, and cognitive exercises has recently grown. In the present review, we aimed to determine whether and under which conditions combined training could be more effective than separated cognitive and physical training, thanks to a structured framework build around seven interacting constructs (stimuli, settings, targets, markers, outcomes, moderators, and mechanisms), which collectively afford a global picture of the determining factors of combined training. We concluded that the general principles underlying the effectiveness of combined training were still difficult identify, due to the heterogeneity of the available studies. However, our analysis also suggested that, when they are well-designed and well-conducted, combined training interventions are more effective than separated physical and cognitive training to improve brain and cognition in older adults. Also, we identified still not answered questions, which could be addressed in futures studies. Finally, we showed that the new categorization of combined training could be also applied to review the literature on training with exergames.

## Introduction

It is now well demonstrated that exercise may attenuate or delay (at least partially) age-related alterations of the various functional subsystems. The question remains, however, of which type(s) of training intervention(s) is/are most effective to improve brain functioning and cognitive performance (e.g., Colcombe and Kramer, 2003; Colcombe et al., 2004; Erickson et al., 2013; Cao et al., 2016). The present review addresses this issue by capitalizing on the growing interest in interventions that combine physical, motor, and cognitive exercises (Pesce and Voelcker-Rehage, 2020).

Several reviews and meta-analyses have previously been dedicated to combined training delivered via conventional interventions that is, those supervised by a coach and that do not use new technologies, but their conclusions were inconsistent (e.g., Lauenroth et al., 2016; Zhu et al., 2016; Gheysen et al., 2018; Guo et al., 2020). A possible reason that may explain these conflicting findings is the lack of a strong conceptual framework to put some order in the available literature, thus preventing to afford a clear picture of the mechanisms and generalizable principles underlying the effectiveness of combined training interventions. By proposing a structured background of concepts and constructs, the present review paper is a step toward the elaboration of such an organizing framework to analyze the findings reported in controlled or randomized trials on conventional combined training interventions. Our aim was threefold. First of all, to determine whether and under which conditions combined training is more effective than separated cognitive and physical training. Secondly, to identify the still unanswered questions and issues that could be addressed in further studies. Thirdly, to propose new directions to inform future research and practical applications.

## A Conceptual Framework for Analyzing Combined Training Interventions

**FIGURE 1:**
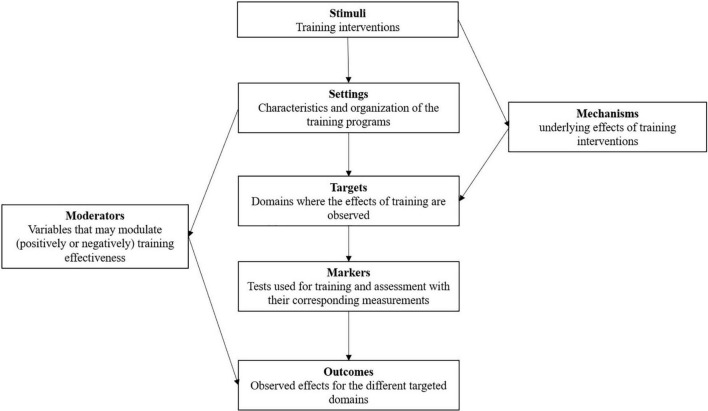
A multi-dimensional analysis of combined training. Detailed explanations are provided in the text.

Based on the existing knowledge on physical, motor and cognitive training, we developed a conceptual structure to articulate seven interacting constructs, which collectively afford a global picture of the determining factors of combined training. Specifically, we distinguished: (1) the stimuli, which refer to the different types of combined training; (2) the settings, which are the organizing features of training programs (frequency, duration, intensity, instructions, feedback, individualization, progressivity of increase in difficulty…), (3) the targets of training, which were limited in the present paper to brain and cognitive levels, but other levels could be added in further works; (4) the markers that is, the tasks/tests used to train/assess the participants, respectively; (5) the outcomes of the different types of training that is, the observed effects at brain and cognitive levels; (6) the moderators that modulated the effects of training, and (7) the potential mechanisms, which were explicitly mentioned in the different studies to predict and/or explain the effects of combined training (see Figure 1).

### Stimuli

Conventionally, training is defined as a set of planned, structured, and repetitive (cognitive, physical, motor, or combined) exercises delivered for a given period (i.e., several weeks or months), to improve the functional capacities of the trained individuals (Caspersen et al., 1985). The different types of combined training interventions under consideration in this review have been identified through a careful analysis of the literature, in order to identify the separated components that were combined and then, how they were combined. For types of training interventions were identified, namely: cognitive training, physical training, muscular resistance training and complex motor skills training.

*Cognitive training* refers to the repeated practice of progressively more difficult exercises designed to stimulate a particular cognitive function or a set of cognitive processes (Bamidis et al., 2014). The cognitive tasks used for training interventions can be implemented either through classic paper and pencil supports or through digital supports presented either on a computer or a tablet (e.g., Anguera and Gazzaley, 2015; Bediou et al., 2018; Dale et al., 2020). *Physical training* refers to body movements that are produced by the contraction of the skeletal muscles and that increase energy expenditure. Endurance training targets predominantly the cardiovascular system, generally through the use of cyclic activities (e.g., walking, running, cycling, and rowing) during long-duration exercises (i.e., >30 min). The outcome of physical training is physical fitness, which is usually assessed in laboratory through aerobic capacities (e.g., VO_2_ max), or indirectly and more globally measured by the distance traveled in different walking field tests (e.g., Simonsick et al., 2001; Chan and Pin, 2019). *Muscular resistance* training generally consists of exercises carried out with body weight, free weights, and/or machines, with load ranging from 30 to 100% of one-repetition maximum. It increases neuromuscular control and muscle force, together with or independent of muscle mass (Clark and Manini, 2010). Thus, the effects of muscular resistance training are generally evaluated by measuring the gain in muscular force (e.g., Van Roie et al., 2013). *Motor training* refers to the practice of complex movements that is, movements that involve the coordination of several degrees of freedom (multi-joint, multi limb…) and require attentional and executive processes to be elaborated and accurately controlled. Thus, complex motor skills training offers a possible bridge between cognitive training and physical exercise (Voelcker-Rehage et al., 2010; Pesce, 2012; Moreau et al., 2015) (summary of separated physical, motor and cognitive training for definitions, effects and underlying mechanisms in Supplementary Table 1).

According to the well-demonstrated effects of separated training, it can be speculated that combining cognitive, motor, and/or physical exercises should be more effective than training interventions carried out in isolation to improve brain and cognition in older adults (Stojan and Voelcker-Rehage, 2019; Pesce and Voelcker-Rehage, 2020; Temprado et al., 2020; Torre et al., 2021). The question remains, however, of how separated training must be combined in the literature to design effective intervention programs. As a prerequisite of our review, we identified the different combinations of physical, motor, and cognitive exercises used in the literature.

#### Different Types of Combined Training Interventions

**FIGURE 2:**
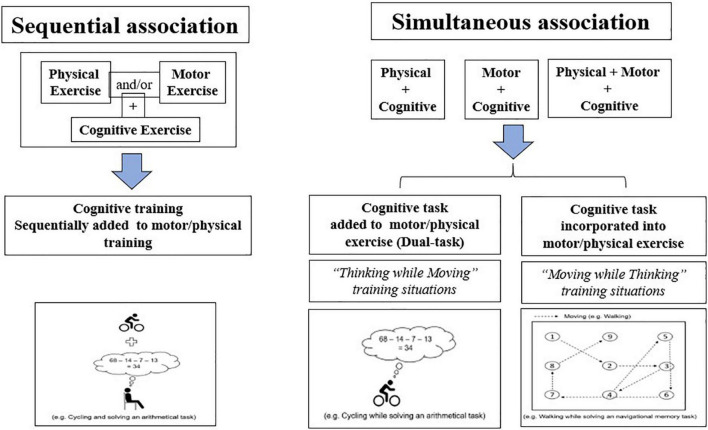
The different combined training interventions (inspired from Herold et al., 2018; Torre et al., 2021).

Three main combinations were identified: (i) physical-cognitive training (PCT), which correspond to the association of endurance (aerobic) and/or muscular resistance training and cognitive training, either sequentially or simultaneously; (ii) motor-cognitive training (MCT), which refers to the association of complex motor skills training and cognitive training implemented through the addition of cognitive tasks separated from the motor task (e.g., mental calculation), and (iii) multi-domain training (MDT), which consists of associating aerobic exercises, complex motor skills, and cognitive tasks through lab-customized training situations. Notably, MDT can also be implemented through natural motor activities (e.g., Tai Chi, Dance, or Nordic Walking) but, in the present review, we limited our analysis to studies in which it was possible to identify the different training components (physical, motor, and cognitive) that were associated with each other. However, as we considered these natural activities as possible supports of MDT, this point will be addressed in the general discussion. Similarly, physical-motor training, which refers to the association of endurance/muscular training and the practice of complex motor skills has not been considered as a combined training intervention. Indeed, although complex motor skill training are themselves hypothesized to cognitive control processes, cognitive exercises were not explicitly identified in the related studies (e.g., Vaughan et al., 2014; Temprado et al., 2019). Within each different type of combined training intervention, two sub-categories were also distinguished, depending on whether cognitive and physical/motor exercises were delivered either sequentially or simultaneously (Tait et al., 2017). Moreover, within the simultaneous association, we distinguished those that associated an additional cognitive task (so-called “Thinking while Moving,” or to dual-task situations) and those where cognitive processes were embedded into complex motor tasks (“Moving while Thinking”) (for a similar distinction, see Herold et al., 2018; Torre et al., 2021) (Figure 2).

### Settings

Through the settings, we identified how the training programs were organized and conducted. It was done with respect to the duration of the training, the frequency, and duration of sessions, the total number of sessions, the intensity of physical exercises, the complexity of the used motor skills and cognitive exercises, their variety, etc. Also, we tried to identify what type of instructions were given, whether and how feedback was given to participants, or whether training loads were individualized and progressively increased.

### Targets

This construct refers to which brain structures and/or functions and cognitive processes were impacted by the different types of combined training. Though it was out of the scope of the present review, other targets (sensorimotor, physiological, biomechanical…) could be considered in future reviews.

### Markers

This construct refers, on the one hand, to the tasks and tests used for training and assessment, respectively, and, on the other hand, the corresponding measurements (e.g., response time, errors…).

### Outcomes

This construct refers to the observed effects of training for the different targeted domains. In the present paper, we limited our analyses to changes in brain and cognition.

### Moderators

This construct refers to the variables that modulate (positively or negatively) training effectiveness. Some of the moderators relate to the settings of training interventions, while others refer to the characteristics of participants (age, education…). An important question was whether specific moderators to combined training could be identified.

### Mechanisms

Mechanisms explain why combined training interventions were (or not) effective, and in particular, more effective than separated training. A general hypothesis is that combined training could allow capitalizing on additive or interactive effects of presumably different/complementary mechanisms related to physical, motor, and cognitive exercises. We tried to identify the frameworks, models, or specific physiological, neurobiological, or psychological mechanisms that were mentioned in the reviewed studies, to make hypotheses and/or to explain the observed results.

## Methods

### Search Strategy

The present review paper is based on a re-analysis of Randomized Controlled Trials (RCT) and Controlled Trials (CT) references in the reviews and meta-analyses dedicated to conventional combined training interventions and published, in English, from 2010 to April 2021. In addition, recently published RCT/CT (from April 2020 to April 2021), not mentioned in the selected reviews, were also identified and screened on the same basis. Thus, a first step consisted of identifying the reviews and meta-analysis of interest, through systematic searches conducted in Medline/Pubmed/Science Direct and Google Scholar Search terms were (“review” OR “meta-analysis”) AND (“combined training” OR “cognitive-motor” OR “dual-task training” OR “multicomponent training” OR “multidomain training”) AND (“older adults” OR “healthy older adults”) AND “English.” After this first step, the different studies cited as references in the reviews were carefully analyzed. Unpublished articles, thesis, dissertations, or book chapters were not considered. Only the studies investing “lab-customized” combined training interventions were included in the analysis that is, reviews, meta-analyses, and studies focusing only on “natural” activities such as Tai Chi, Dance, or Nordic walking were not included in the analysis. Then, a second step consisted of identifying the studies published between November 2020 and August 2021 through similar systematic searches (“combined training” OR “cognitive-motor” OR “dual-task training” OR “multicomponent training” OR “multidomain training”) AND (“older adults” OR “healthy older adults” AND “English”). Only one study (Jardim et al., 2021, published in February 2021) met the inclusion criteria.

### Selection Process and Data Extraction

**FIGURE 3:**
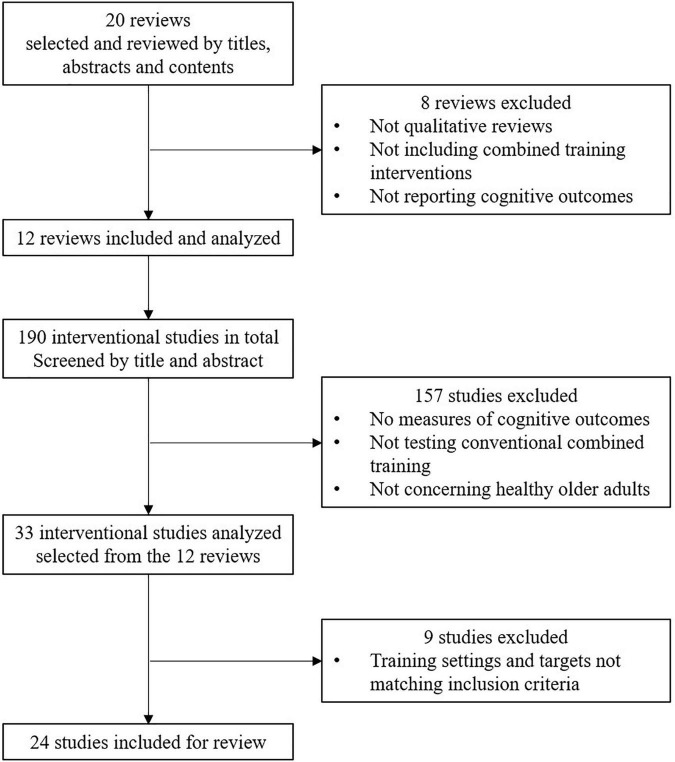
Flow chart of the selection process.

The screening of the papers identified after the systematic searches was made by title, abstract and content relevance by 2 reviewers (MMT and JJT). Twenty reviews were first identified on this basis. Then, after a careful analysis, reviews and meta-analyses were considered relevant if they met the following criteria: (i) concerning one or several types of the combined training types identified in our framework, (ii) to be delivered through conventional training (i.e., excluding exergames), (iii) including only healthy older adults (>60 years old) or a separated analysis of healthy older adults among other populations. Finally, twelve reviews and meta-analyses were finally included in the analysis, based on these criteria (see Supplementary Table 1). The next step then consisted of selecting the most relevant studies among the references cited in the selected reviews and meta-analyses. To be included, Randomized Controlled Trials (RCT) and Controlled Trials (CT) had: (i) to consist in a training program lasting at least 4 weeks, (ii) to include one session of 30 min or more per week, (iii) to report measures of executive functions and/or attention and/or memory and/or information’s processing speed. The lack of measurement of physical (VO_2_, muscular strength…) and behavioral outcomes (balance control, walking speed, motor coordination…) was not considered a reason for exclusion. On the other hand, we did not include the studies in which only cognition in everyday life, motivation, well-being, stress, depression, or anxiety were measured. Studies reporting analyzes of brain activity were considered relevant only if they concomitantly reported cognitive outcomes. Studies that only consisted of dual-task cost measured during gait, but did not include separated measures of cognitive processes (EF, attention, memory, …), were considered marginally relevant and then, not included. Finally, over the 190 referenced studies, 34 studies were identified as possible candidates for the analysis, while 25 were finally selected for review (see Figure 3 and Supplementary Table 1). Since the studies were selected from already existing reviews, we did not repeat the process of quality assessment.

## Review of Conventional Combined Training Studies

### Physical-Cognitive Training

#### Stimuli

Of the 24 studies selected for the analysis, 11 concerned physical–cognitive training. Seven studies included four groups: physical-cognitive training (PCT), cognitive training, physical training, and a control group (Fabre et al., 2002; Legault et al., 2011; Shatil, 2013; Linde and Alfermann, 2014; McDaniel et al., 2014; Shah et al., 2014; Desjardins-Crepeau et al., 2016). They all consisted of sequential training, with physical exercises presented before cognitive exercises, except in Legault et al. (2011). The four other studies tested simultaneous training programs (Theill et al., 2013; Eggenberger et al., 2015a; León et al., 2015; Norouzi et al., 2019) and did not include four groups: 3 of them compared the PCT group with a control group (Theill et al., 2013; León et al., 2015; Norouzi et al., 2019), 2 with a physical training group (Eggenberger et al., 2015a; León et al., 2015) and 1 with a cognitive training group (Theill et al., 2013). Notably, Eggenberger et al. (2015a) also included an additional group of participants who practiced exergaming (i.e., a dance video game), while Norouzi et al. (2019) only compared two dual-task training programs involving the (simultaneous) association of physical-cognitive and physical-motor exercises, respectively, in addition to an inactive control group. Finally, 5 studies on simultaneous training were considered after including Eggenberger et al. (2015b), who published additional analyses of the data recorded during their initial experiment (Eggenberger et al., 2015a).

#### Settings

In all the studies, pre-and post-intervention assessments of performance were used. Only Shah et al. (2014) additionally tested performance at the midpoint of the training program. The permanence of the effects was investigated in 2 studies (Linde and Alfermann, 2014; Norouzi et al., 2019), in which participants were re-tested in 3 months or 1 year after the end of the training period.

In the studies on sequential training, physical training consisted of aerobic exercise alone (Fabre et al., 2002; Legault et al., 2011; McDaniel et al., 2014) or in association with muscular resistance exercises (Linde and Alfermann, 2014; Shah et al., 2014). The prominent support of physical training was walking/running (Fabre et al., 2002; Legault et al., 2011; Linde and Alfermann, 2014; McDaniel et al., 2014; Shah et al., 2014; Desjardins-Crepeau et al., 2016), while one study used cycling (Legault et al., 2011), and another, full-body movements (Shatil, 2013). In all the studies, physical exercises were similar for PCT and physical training alone. Similarly, the cognitive exercises used for PCT were the same as those used for cognitive training. The supports of cognitive exercises were either paper and pencil (Fabre et al., 2002; Linde and Alfermann, 2014), computer (Legault et al., 2011), or brain games (Shatil, 2013; Shah et al., 2014).

The supports of physical training differed between the 4 studies on simultaneous training, that is, either walking (Theill et al., 2013; Eggenberger et al., 2015a), resistance training of lower limbs on an isokinetic exercise device (Norouzi et al., 2019), or various full-body movements (walking dance and muscular resistance; León et al., 2015). The cognitive exercises used for combined training were displayed in the form of dual-task situations. They were either working memory tasks displayed on a monitor (Theill et al., 2013; Eggenberger et al., 2015a) or a battery of (12) cognitive tests (Norouzi et al., 2019). Notably, in addition to classic cognitive-motor dual-task situations, Norouzi et al. (2019) used a motor-motor dual task by performing a cognitively demanding motor task during muscular resistance training (i.e., throwing a bag, holding a medicine ball in both hands…). Then, they compared the two forms of PCT.

#### Targets

The targets of interest were either global cognition or/and specific cognitive processes. All the studies tested one (e.g., Fabre et al., 2002; Norouzi et al., 2019) up to 17 cognitive abilities (e.g., Shatil, 2013). The most frequently tested abilities were memory (Fabre et al., 2002; Legault et al., 2011; Shatil, 2013; McDaniel et al., 2014; Shah et al., 2014; Eggenberger et al., 2015a), executive functions (Fabre et al., 2002; Legault et al., 2011; Shatil, 2013; McDaniel et al., 2014; Shah et al., 2014; Eggenberger et al., 2015a; León et al., 2015), attention (Shatil, 2013; Theill et al., 2013; Shah et al., 2014; Eggenberger et al., 2015a) and information processing speed (Shatil, 2013; Theill et al., 2013; Shah et al., 2014; Eggenberger et al., 2015a; Norouzi et al., 2019). In some studies, scores were calculated for global cognition by clustering the results observed for the different targeted processes (see Shatil, 2013, for details). Brain activity was recorded in one study (Shah et al., 2014). Some studies reported changes in physical fitness induced by training (e.g., Fabre et al., 2002; Legault et al., 2011; Linde and Alfermann, 2014; McDaniel et al., 2014; Shah et al., 2014), but correlations between improvements in physical fitness and cognition were not systematically investigated.

#### Markers

A large variety of different tasks were used across the reviewed studies, even to test similar cognitive functions. The most frequently used tests were the Wechsler Test set (different parts to test different functions) (Fabre et al., 2002; Eggenberger et al., 2015a; Desjardins-Crepeau et al., 2016), the Trail Making Test (A-B) (Fabre et al., 2002; Linde and Alfermann, 2014; McDaniel et al., 2014; Eggenberger et al., 2015a), the Rey Auditory Verbal Learning test (Shah et al., 2014), and the Stroop test (McDaniel et al., 2014). In 2 studies (Shatil, 2013; Theill et al., 2013), the tests used for cognitive training and cognitive assessment were slightly different (to avoid test/re-test effects). Fitness level was assessed in few studies through an incremental test of maximal aerobic capacity on a cycloergometer (Fabre et al., 2002), or different walking tests (Legault et al., 2011; Linde and Alfermann, 2014; Shah et al., 2014). Notably, no study on simultaneous training assessed physical fitness outcomes.

#### Moderators

Only healthy older adults were considered in the selected studies. The age of participants ranged from 50 years up to 93 years. Though age could be considered an indirect proxy of functional capacities, this factor was not used as a moderator in the analyses carried out in the different studies, even in those in which the age range of participants was very large (e.g., 35 years). Education level was reported in only one study (McDaniel et al., 2014).

In sequential training studies, the order of presentation and time delay between physical and cognitive training could be considered as a specific moderator of effectiveness (see Tait et al., 2017). However, only three studies mentioned this information, and time delay was never systematically manipulated. In five studies, physical and cognitive training were carried out within a day (Legault et al., 2011; Shatil, 2013; Shah et al., 2014), while in three studies, they were performed in separated days (Fabre et al., 2002; Linde and Alfermann, 2014; McDaniel et al., 2014). In two studies, physical training was carried out before cognitive training, within the same day (Legault et al., 2011) or on different days (Linde and Alfermann, 2014).

The sessions of physical training lasted between 20 and 90 min, while those of cognitive training were quite similar (i.e., between 30 and 90 min). For all the studies, the frequency of sessions was 2 or 3/week for physical training and between 1 and 5 sessions/week for cognitive training. Then, depending on the length of the training program, the total number of training sessions was between 12 up and 72 for physical training, and between 8 and 80 for cognitive component training. Due to the variety of the length of training programs (4, 8, 10, 12, and 16 weeks up to 6 months), the frequency of sessions per week, and the duration of sessions in the different studies, it was quite impossible to identify a typical combined training program. Among the three studies on simultaneous training, the duration of the training session was between 40 and 80 min, with a frequency of 2 or 3 sessions/week. The total number of sessions was between 12 and 24, which was much less than in studies on sequential training (Theill et al., 2013; León et al., 2015; Norouzi et al., 2019).

Attendance to training programs was generally high in all studies (>80%). Notably, the distinction between responders–non-responders to training programs was never considered in the reviewed studies, although it might significantly affect the interpretation of the results (see Temprado et al., 2019, for an illustrative example).

#### Outcomes

##### Physical-Cognitive Training Versus Control Group

Seven studies on sequential training (Fabre et al., 2002; Legault et al., 2011; Shatil, 2013; Linde and Alfermann, 2014; McDaniel et al., 2014; Shah et al., 2014; Desjardins-Crepeau et al., 2016) and 4 on simultaneous training (Theill et al., 2013; Eggenberger et al., 2015a; León et al., 2015; Norouzi et al., 2019) assessed the effectiveness of PCT relative to a control group (i.e., inactive or practicing stretching exercises). All the studies, except Legault et al. (2011), reported significant differences between pre-and post-tests in at least one cognitive function. Significant improvements were reported for (different forms of) memory (Fabre et al., 2002; Shatil, 2013; McDaniel et al., 2014; Shah et al., 2014; Norouzi et al., 2019), paired associated learning (Fabre et al., 2002), information processing speed (Shatil, 2013; Linde and Alfermann, 2014), visual scanning and naming (Shatil, 2013) and executive control (Theill et al., 2013), including inhibition and task-switching processes (Desjardins-Crepeau et al., 2016). The inconsistent results observed by Legault et al. (2011) might be explained by the rather short length of the cognitive training program (12 min/day, 2 days/week over 8 weeks). They might be also the consequence of the presence of cognitive training before physical training, while a reverse order was adopted in most sequential studies. Only two studies included a follow-up to test the permanence of training effects. They reported either no (McDaniel et al., 2014) or weak (Eggenberger et al., 2015a) maintenance of effects on the tested functions. McDaniel et al. (2014) attributed these results to the lack of similarity between the training and assessed functions, respectively, while Eggenberger et al. (2015a, p. 1,345) stated that the possible effect of additional cognitive training during the follow-up period was plausible but impossible to estimate.

##### Physical-Cognitive Training Versus Separated Physical Training

Nine studies, 7 on sequential training (Fabre et al., 2002; Legault et al., 2011; Shatil, 2013; Linde and Alfermann, 2014; McDaniel et al., 2014; Shah et al., 2014; Desjardins-Crepeau et al., 2016) and 2 on simultaneous training (Eggenberger et al., 2015a; León et al., 2015), compared PCT and separated physical training. Conflicting results were observed. Indeed, in three studies, (sequential) PCT was not found more effective than physical training carried out in isolation to improve cognitive performance (Legault et al., 2011; Linde and Alfermann, 2014; Shah et al., 2014). In the 6 other studies (5 sequential and 1 simultaneous), larger benefits were observed for PCT, at least for one cognitive function (Fabre et al., 2002; Shatil, 2013; McDaniel et al., 2014; Eggenberger et al., 2015b; León et al., 2015; Desjardins-Crepeau et al., 2016). Importantly, among these studies, cognitive performance increased after separated physical training in only 4 of them (Fabre et al., 2002; Eggenberger et al., 2015a; León et al., 2015; Desjardins-Crepeau et al., 2016). On the other hand, in the studies in which no differences between PCT and separated physical training were found, the effects of separated physical training on cognitive performance were weak or even absent, as attested by the lack of difference between physical training groups and control groups (Legault et al., 2011; Linde and Alfermann, 2014; McDaniel et al., 2014; Shah et al., 2014).

##### Physical-Cognitive Training Versus Separated Cognitive Training

Seven studies compared PCT and cognitive training (Fabre et al., 2002; Legault et al., 2011; Shatil, 2013; Theill et al., 2013; Linde and Alfermann, 2014; McDaniel et al., 2014; Shah et al., 2014). In four studies, cognitive performance improved after both PCT and separated cognitive training (Fabre et al., 2002; Shatil, 2013; Theill et al., 2013; McDaniel et al., 2014). Among them, larger benefits of PCT were only observed in two studies, one that implemented sequential training (Fabre et al., 2002) and another using simultaneous training (Theill et al., 2013). Notably, Fabre et al. (2002) did not observe a post-intervention difference between separated physical and cognitive training, while their combination led to larger, though under-additive, benefits than those observed after both separated interventions (physical-cognitive training: Δ = +12%; separated cognitive training: Δ = +8%; separated physical training: Δ = +7%). Theill et al. (2013) did not include a physical training group so that the weight of the increase in cognitive performance induced by physical training within the combined training intervention was impossible to estimate.

##### Comparison of Two Different Forms of Physical-Cognitive Training

Norouzi et al. (2019) compared motor-cognitive and motor-motor dual-task training found that both training modes allowed to improve cognitive performance, though benefits were larger for the motor-cognitive training (MCT) program.

#### Mechanisms

Before considering the mechanistic explanations that were provided in the different studies to account for the observed results (for details, see Section “Discussion”), as a prerequisite, we aimed to identify the studies in which a theoretical framework was explicitly mentioned, either in the introduction (to build the operational hypotheses), or in the discussion (to explain the observed results).

In general, very few studies were explicitly and firmly grounded on theoretical bases. Moreover, very few papers mentioned a specific conceptual framework or specific mechanisms that could be related to the combination of physical and cognitive training (i.e., not only related to separated training), to strengthen their hypotheses or to explain their results (Linde and Alfermann, 2014; McDaniel et al., 2014; Eggenberger et al., 2015a; Desjardins-Crepeau et al., 2016). In particular, no prediction was made in the different studies about the possible under- or over-additive effects of combined training relative to the sum of the effects of separated training.

The most frequently cited framework was the (very general) cognitive enrichment hypothesis, which assumes that the behaviors of an individual (including cognitive activity, physical exercise, etc.) have a positive impact on cognitive functioning in old age (e.g., Linde and Alfermann, 2014; Eggenberger et al., 2015a). The cardiovascular fitness hypothesis, which predicts that physical activity exerts its positive influence on cognition through the improvement of cardiovascular fitness was scarcely mentioned (e.g., Linde and Alfermann, 2014). Eggenberger et al. (2015a) hypothesized that: (i) separated physical and cognitive training might impact different cognitive processes, and (ii) physical training might lead to more general effects on brain plasticity than cognitive training. However, nothing in their findings allowed them to confirm this assumption.

At a neurobiological level, the neurotrophic factors hypothesis was frequently mentioned, either to build hypotheses about brain plasticity resulting from the different types of training (Theill et al., 2013; Linde and Alfermann, 2014) or to explain the observed results (Eggenberger et al., 2015a; Norouzi et al., 2019). Three studies (Shatil, 2013; Theill et al., 2013; Linde and Alfermann, 2014) explicitly elaborated their hypotheses based on this framework. However, whether and how brain plasticity was specifically/differently affected by each separated intervention and by their combination was not addressed in the different studies. Increase in blood flow and oxygen perfusion, which are associated with activation of glucose metabolism were frequently mentioned to explain the improvement of cognitive performance resulting from physical activity (e.g., Fabre et al., 2002; Shah et al., 2014; Eggenberger et al., 2015a). However, only the results observed by Shah et al. (2014) corroborated this hypothesis by analyzing brain activation and blood flow. In the other reviewed studies, no strong predictions or detailed explanations of the results were provided.

Notably, though several studies implemented simultaneous PCT, the potential mechanisms underlying dual-task training were scarcely detailed. At least, it was briefly mentioned that DT interventions might enhance divided attention (Eggenberger et al., 2015a,b; Norouzi et al., 2019), improve task-switching (Eggenberger et al., 2015a,b), or lead to engaging higher brain centers, thereby leading to improvement in cognitive capacities (Norouzi et al., 2019). The frameworks of single channels or multiple resources were scarcely mentioned in the reviewed studies, although they are currently taken as reference models in the DT literature (Wollesen and Voelcker-Rehage, 2013; Schaefer, 2014; Wollesen et al., 2020).

#### Discussion

Several important conclusions can be drawn from the reviewed studies on PCT. First of all, the comparison with control groups suggested that PCT allowed improving different cognitive functions, such as (the different forms of) memory, paired associated learning, information processing speed, visual scanning, and naming, verbal fluency, attention and executive control, including task-switching and inhibition. However, due to the heterogeneity of the targeted processes, it was difficult to estimate: i) whether some cognitive functions were most sensitive to PCT than to separated training, and ii) whether a larger number of functions were affected by PCT than by separated physical or cognitive training. Though critical to better understanding the underlying mechanisms of combined training, these issues were never specifically addressed in the reviewed studies.

The effects of PCT on cognition were observed for both sequential and simultaneous training programs, whatever the age or education of (healthy older) participants (from 50 up to 93), under the reserve that the training duration was long enough (from 30 up to 90 sessions) and training programs well designed (e.g., individualized; Fabre et al., 2002). In this respect, it has been hypothesized that simultaneous training could be more effective than sequential training (Desjardins-Crepeau et al., 2016), but this issue was not addressed in the available studies. In most studies on sequential training, physical training was presented before cognitive training, which seemed to be more effective than the inverse (for supporting evidence, see Legault et al., 2011). Finally, no firm conclusion can be drawn about the permanence of combined training effects and whether the effects of PCT were generalizable (i.e., transferable) to untrained tasks or, even more, transferable than separated physical and cognitive training, remains to be established (see for a discussion, McDaniel et al., 2014; Desjardins-Crepeau et al., 2016).

As exercise is better than inactivity, the effects of PCT relative to control groups were not surprising. On the other hand, a less trivial finding was that the superiority of PCT over either separated cognitive or physical training was not firmly established. Several reasons can explain this observation. First of all, only seven studies (all implementing sequential training) included four experimental groups. Secondly, among these studies, few of them met the criteria (in our view required) to firmly demonstrate the superiority of PCT over separated training interventions, that is: (i) a post-intervention increases in cognitive performance for both separated physical and cognitive training relative to a control group, and (ii) a significant post-intervention difference between PCT and separated training for cognitive performance. Actually, among the seven studies that included four experimental groups, two did not report a significant post-intervention difference between PCT and separated cognitive or physical training (e.g., Legault et al., 2011; Desjardins-Crepeau et al., 2016) while, finally, only one met all the above criteria (i.e., Fabre et al., 2002) and supported the superiority PCT over separated training. Interestingly, the results observed by Fabre et al. (2002) suggested slight under-additive post-intervention effects of combined training on memory quotient relative to the sum of the respective effects of separated cognitive and physical training (i.e., 12% vs. 15%). However, this conclusion cannot be generalized, since no more studies are allowed to carry out this analysis.

Notably, several studies that did not include four experimental groups nevertheless concluded that PCT had larger beneficial effects than separated training. This was the case when a significant post-intervention difference was observed between the PCT group and the separated cognitive and/or physical training groups, even if the effects of separated interventions were weak or not significant that is, equivalent to a control group. For instance, in few studies, the effects of physical training on cognitive functions were not significant, which could suggest that the intensity of physical training was too low to be effective. However, instead of considering this interpretation, the authors rather concluded that additional cognitive training was necessary to potentiate the effects of physical effort (e.g., Shatil, 2013; Linde and Alfermann, 2014; McDaniel et al., 2014; Desjardins-Crepeau et al., 2016). In our view, this interpretation was speculative but it is fair to mention that it is plausible and consistent with those provided elsewhere in the exercise and cognition literature (Pesce, 2012; Diamond and Ling, 2016, 2020; Pesce and Voelcker-Rehage, 2020).

In summary, although the superiority of PCT over separated training is often taken for granted in the related literature, until now, very few studies have firmly confirmed this hypothesis, principally due to the small number of well-conducted studies, rather than to the lack of theoretical foundations in favor of combined training. Specifically, only two studies fully supported the superiority of PCT over separated training that is, Fabre et al. (2002, for sequential training) and, to a lesser extent, by Theill et al. (2013, for simultaneous training). This suggests that when physical and cognitive exercises were well-designed and well-conducted, both their sequential and simultaneous association into PCT potentiated their separated effects. However, in most studies, it was difficult to assess the quality of the training programs since the details of exercises and procedures (e.g., individualization, progressive increase in difficulty…) were not provided, in particular concerning cognitive training. More generally, among the reviewed studies, few, if any, were based on the training principles previously established by sport sciences. Moreover, no one used multi-domain cognitive training, which is widely recognized as the most effective training strategy to improve brain and cognition, independent of its combination with physical exercises (e.g., Cheng et al., 2012). Moreover, even when convincing evidence supporting the superiority of PCT over separated training was provided, reference to the underlying mechanisms remained elusive. Finally, it can be concluded that PCT may be more effective than separated training but further (four arms) studies are needed to confirm this hypothesis and, in particular, to identify the mechanisms that underlie the specificity of combined training relative to separated training.

### Motor-Cognitive Training

#### Stimuli

Of the four motor–cognitive training studies that have been selected, only one implemented sequential training (Oswald et al., 2006), while the three others used simultaneous training (Marmeleira et al., 2009; Hiyamizu et al., 2011; Falbo et al., 2016). The sequential training study compared four groups – a MCT group, a motor training group, a cognitive training group, and an active control group – while of the three studies on simultaneous training, two compared MCT and physical training (Hiyamizu et al., 2011; Falbo et al., 2016), while the other assessed the effects of MCT relative to the baseline performance of the group before training (Marmeleira et al., 2009).

#### Settings

In the four analyzed studies, motor training incorporated challenging activities that engaged the participants to mobilize several types of motor skills: coordination (e.g., walking with arms circles), balance (e.g., maintaining a unimodal stance with and without swinging the free leg), squatting while extending an elastic band with arms, walking through an agility ladder at a different speed, dancing, or simple structured game skills in which aerobic effort and muscular resistance were only minimally required. Cognitive training aimed to stimulate various cognitive functions (see Section “Targets”) through classic paper and pencil exercises. Similar cognitive and motor exercises were used for combined and separated training interventions. In the sequential training study, motor training was carried out before cognitive training (Oswald et al., 2006). MCT consisted of adding cognitive exercises to complex motor tasks (i.e., dual-task situations), which allowed to target executive functions (e.g., planning and inhibitory control), perceptual discrimination, attention, memory, or response inhibition and switching (for illustrative examples of exercises, see Marmeleira et al., 2009, Supplementary Table 2).

#### Targets

Whatever the type of combination (i.e., sequential/simultaneous), cognitive functions were primarily targeted and, secondarily, behavioral control mechanisms. All the four studies tested at least one cognitive function. In the sequential study (Oswald et al., 2006), speed of information processing, attention, memory, and reasoning were assessed. In the three studies on simultaneous training, visual information processing speed, divided attention, selective attention, and executive functions were tested (Marmeleira et al., 2009; Hiyamizu et al., 2011; Falbo et al., 2016). The assessed behavioral outcomes were different in the three studies: driving performance in a simulator (Marmeleira et al., 2009), balance control in a dual-task situation (Hiyamizu et al., 2011), and gait pattern in a dual-task situation (Falbo et al., 2016).

#### Markers

In general, numerous tests were used to assess cognitive functions. For instance, in the study on sequential training, 13 tests were used [e.g., Digit symbol substitution test (DS-G), Memory span test (Wechsler adults intelligence scale; see Oswald et al., 2006; Supplementary Table 2 for details)]. In the three studies on simultaneous training, the Trail Making Test A and B (Marmeleira et al., 2009; Hiyamizu et al., 2011), the Stroop color-word test (Marmeleira et al., 2009) or a Random Number Generation (RNG) task were used. The RNG task aimed to calculate various indices of executive functions, inhibition, and working memory (see Falbo et al., 2016, for details). In two studies on simultaneous training, single and dual-task situations were used to assess cognitive-motor processes: upright standing on a force platform (balance control) in association with a Stroop task (Hiyamizu et al., 2011) or walking while performing a Random number generation test (Falbo et al., 2016). Changes in dual-task costs were calculated as markers of training effects in the cognitive domain. All four studies assessed motor capacities (whole-body coordination, flexibility, static balance, and mobility) through different tests (Oswald et al., 2006; Marmeleira et al., 2009; Hiyamizu et al., 2011; Falbo et al., 2016).

#### Moderators

The age of the participants ranged from 65 years up to 93 years. No study proposed a stratification into age groups. All the studies measured the short-term effects of intervention (Marmeleira et al., 2009; Hiyamizu et al., 2011; Falbo et al., 2016). In addition, Oswald et al. (2006) also assessed long-term effects (5 years). In the sequential training study, physical and cognitive training were carried out the same day and motor training was presented before cognitive training. Both motor training and cognitive training sessions lasted 45 min so that the combined training session lasted 90 min. The training programs were carried out over 12 months, 1 session/week for a total of 52 sessions. In the simultaneous training studies, the session lasted about 1 h and was carried out 1–3 times a week. Thus, the total number of sessions were ranged from 24 up to 36 (for further details, see Marmeleira et al., 2009; Hiyamizu et al., 2011; Falbo et al., 2016).

#### Outcomes

##### Motor-Cognitive Training Versus Control Group

Three studies (out of 4) testing MCT relative to control groups have reported significant improvement in at least one cognitive function after intervention (Oswald et al., 2006; Marmeleira et al., 2009; Falbo et al., 2016). Oswald et al. (2006) found significant differences between the MCT group and the control group for information processing speed, attention, and memory. Progress in cognitive performance paralleled increases in physical fitness, though no details were provided about the improved domains (i.e., strength, endurance, coordination, flexibility, and balance). On the other hand, Marmeleira et al. (2009) reported the benefits of MCT for both cognitive (visual attention, executive functioning, and information processing speed) and physical (strength, cardiorespiratory endurance, flexibility, and balance) functions. Falbo et al. (2016) observed a marginal improvement of inhibitory performance after MCT, which was paralleled by a slight increase in walking performance in flat walking conditions but not in conditions of walking in an obstacle-cluttered environment. In dual-task situations, a significant increase in Stroop task performance has been reported by Hiyamizu et al. (2011) and a shorter reaction time by Marmeleira et al. (2009).

##### Motor-Cognitive Training Versus Physical Training

Among the three studies (one sequential and two simultaneous) that compared the effects of MCT and physical training on cognitive functions, a slight difference was observed, thereby supporting larger benefits of combined training (Oswald et al., 2006; Hiyamizu et al., 2011; Falbo et al., 2016). Notably, physical training improved cognitive performance in only one study (Oswald et al., 2006).

##### Motor-Cognitive Training Versus Cognitive Training

In the only one study that compared MCT and cognitive training (Oswald et al., 2006), significant benefits were reported for memory, attention, information processing speed, and reasoning after both motor-cognitive and cognitive training interventions. Moreover, a significant difference was observed between the MCT and the cognitive training groups.

#### Mechanisms

In the four studies considered, no detailed explanation was provided neither to predict nor to explain the mechanisms underlying the observed results. In their introduction, Oswald et al. (2006) mentioned existing empirical evidence relative to brain plasticity, angiogenesis, and synaptogenesis. In the discussion, the authors hypothesized that the benefits of combined training on cognitive functions resulting from increased metabolic activity could only be exploited if brain cells are challenged in the context of specific cognitive effort. They concluded that only a stimulating learning environment could facilitate the development of new neuronal cells. The remaining three studies (on simultaneous training), were descriptive and did not referred to underlying mechanisms to explain the benefits eventually resulting form of dual-task training (Marmeleira et al., 2009; Hiyamizu et al., 2011; Falbo et al., 2016).

#### Discussion

Due to the small number of studies that investigated MCT and the heterogeneity of their design, it remains difficult to draw generalizable conclusions. The studies that compared MCT and control groups suggested that it was effective to improve cognitive functions, such as visual attention, executive functioning, and information processing speed. Relative to physical training, only slight differences in favor of MCT were reported in the different studies. However, only one study (Oswald et al., 2006) did lend credence to the superiority of MCT over both separated physical and cognitive training. Indeed, it was the only study in which both separated training positively impacted cognitive performance, while differences in favor of MCT were observed. Only one study tested the effects of training during follow-up, showing that the MCT group maintained cognitive benefits of training after 5 years (Oswald et al., 2006). Notably, in all the reviewed studies, the underlying mechanisms that might explain the observed results were not developed. At least, a larger activation brain cortex was expected as a result of motor skill training (see Voelcker-Rehage et al., 2010, 2011, for consistent results in this respect), but whether there was a release of neurotrophic factors (BDNF and IGF-1) in the brain after motor and MCT is still unknown and can only be hypothesized.

In summary, according to these findings, it can be concluded that MCT might potentially lead to larger benefits than separated motor skills training, in particular when cognitive exercises are presented sequentially after physical training (Oswald et al., 2006). However, further studies are necessary to confirm this hypothesis.

### Multi-Domain Training

#### Stimuli

Six studies on multi-domain training (MDT) were selected, but 8 studies were considered in our review since, based on the initial protocol published by Rahe et al. (2015a), additional analyses of the data were subsequently published (Rahe et al., 2015b; Kalbe et al., 2018). Sequential training was implemented in three studies (Pieramico et al., 2012; van het Reve and de Bruin, 2014; Rahe et al., 2015a) and simultaneous training in the three remaining ones (Nishiguchi et al., 2015; Yokoyama et al., 2015; Ansai et al., 2017). Among the three studies on sequential training, the effectiveness of MDT was assessed relative to: (i) a control group (Pieramico et al., 2012), (ii) a physical training group (van het Reve and de Bruin, 2014), or (iii) a cognitive training group (Rahe et al., 2015a). Thus, the additional papers published by Rahe et al. (2015b) and Kalbe et al. (2018) also concerned the comparison of sequential MDT and cognitive training. In the three studies on simultaneous training, MDT was only compared to a control group (Nishiguchi et al., 2015; Yokoyama et al., 2015; Ansai et al., 2017).

#### Settings

Multi-domain training was generally implemented through a combination of aerobic exercise (e.g., walking) and muscular resistance training, together with the practice of complex motor skills (e.g., balance control, dancing, throwing balls to targets, and stepping tasks). However, the details of the used exercises were scarcely provided in the different studies. Physical and cognitive training were conducted either in the same session (Pieramico et al., 2012; Rahe et al., 2015a) or during separate sessions (van het Reve and de Bruin, 2014). Within the studies on sequential training, physical and cognitive exercises were carried out during the same day (Pieramico et al., 2012; Kalbe et al., 2018), physical training was carried out either before (Pieramico et al., 2012) or after cognitive training (Kalbe et al., 2018). In the other study, it was not specified (van het Reve and de Bruin, 2014). Cognitive exercises used in the sequential MDT were either proposed through a paper and pencil support (e.g., crossword, sudoku, and puzzle; Pieramico et al., 2012) or through computerized software (e.g., Cognipluset al., 2014; Neurovitalis et al., 2015a). Within the studies on sequential training, when MDT was compared to either physical training or cognitive training, the physical and cognitive exercises used for MDT and separated training were similar (Pieramico et al., 2012; van het Reve and de Bruin, 2014; Rahe et al., 2015a). Simultaneous training consisted of adding various cognitive tasks (counting down, verbal fluency…) to physical/motor exercises through dual-task situations (see Jardim et al., 2021, Supplementary Table 1, for illustrative examples). Pre-intervention and post-intervention tests were administered in all the studies. One study tested a 1-year follow-up (Kalbe et al., 2018).

#### Targets

All the studies tested at least one cognitive function. Independent of the type of MDT (i.e., sequential or simultaneous), the main targets of training were global cognition (Pieramico et al., 2012; Nishiguchi et al., 2015; Yokoyama et al., 2015; Ansai et al., 2017; Kalbe et al., 2018), memory (Rahe et al., 2015a; Yokoyama et al., 2015; Kalbe et al., 2018), verbal fluency (Pieramico et al., 2012; Rahe et al., 2015a), visuospatial capacities (Pieramico et al., 2012; Yokoyama et al., 2015; Ansai et al., 2017), and executive functions (Pieramico et al., 2012; van het Reve and de Bruin, 2014; Nishiguchi et al., 2015; Yokoyama et al., 2015; Kalbe et al., 2018). Changes in brain structure or functions were assessed in two studies (Pieramico et al., 2012; Nishiguchi et al., 2015), as well as motor behavior (van het Reve and de Bruin, 2014) and motor/physical fitness (Yokoyama et al., 2015). Neurobiological mechanisms were targeted in three studies (Pieramico et al., 2012; Yokoyama et al., 2015; Kalbe et al., 2018).

#### Markers

The most frequently used tests to assess cognition were the MMSE (Pieramico et al., 2012; Nishiguchi et al., 2015; Yokoyama et al., 2015; Ansai et al., 2017), the clock drawing test (Pieramico et al., 2012; Ansai et al., 2017), TMT(A-B) (Pieramico et al., 2012; van het Reve and de Bruin, 2014; Eggenberger et al., 2015a; Nishiguchi et al., 2015; Yokoyama et al., 2015) and the Wechsler scale (Nishiguchi et al., 2015; Kalbe et al., 2018). Dual-task costs during walking or psychomotor reaction time were also assessed (van het Reve and de Bruin, 2014; Yokoyama et al., 2015; Ansai et al., 2017; Jardim et al., 2021). More attention to physical fitness (i.e., cardiorespiratory fitness, lower limbs strength, and agility), balance (single-leg standing), and mobility (maximal step length, Timed Up and Go, and Sit to Stand tests) were given in all the reviewed MDT studies than in PCT and MCT studies. Few studies analyzed peripheral blood levels of neurotrophic factors (APoE, IGF-1, BDNF, and VEGF), dopamine-related genes, or plasma amyloid peptides (Pieramico et al., 2012; Yokoyama et al., 2015; Kalbe et al., 2018).

#### Moderators

The age of the participants ranged from 59 years up to 85 years old. The widest range of age was 35 years. In the studies on sequential training, the physical training sessions lasted at least 40 min to a maximum of 90 min, with 2 to 3 sessions/week. The cognitive training sessions lasted at least 10 min up to 60 min, 2–3 times/week. Due to the heterogeneity of duration and session frequency in the different protocols, the total number of training sessions varied from 14 up to 72 for the physical training component, and from 14 up to a maximum of 90 for the cognitive component training. Within the studies on simultaneous training, training sessions lasted at least 50 min up to 90, with 1–3 training sessions/week. The total number of sessions thus varied from 12 to 36 (Nishiguchi et al., 2015; Yokoyama et al., 2015; Ansai et al., 2017). Also, Kalbe et al. (2018) showed that lower baseline performance, lower educational level, lower blood level of BDNF, and higher blood level of IGF-1 were strong moderators and predictors of MCT effectiveness.

#### Outcomes

##### Multi-Domain Training Versus Control Group

Among the studies that compared MDT to a control group (or to their pre-training baseline), 2 reported significant improvement in, at least, one cognitive function (Pieramico et al., 2012; Nishiguchi et al., 2015). The benefits of MDT were principally observed for memory (Pieramico et al., 2012; Nishiguchi et al., 2015), executive functions (Nishiguchi et al., 2015), and attention (Jardim et al., 2021). On the other hand, Ansai et al. (2017) did not observe any differences with the control group for cognitive functions, after a 12 weeks simultaneous training program (three times/week). It might be attributed to the tests used for cognitive assessment (MMSE, MoCA, and the Clock Drawing test), which did not distinguish the different potentially impacted processes (e.g., attention, executive functions…). In support of this hypothesis, Pieramico et al. (2012) did not observe any differences between the MDT and the control groups for MMSE, after 6 months of sequential training (three times/week of physical/motor training and five times/week of cognitive training).

##### Multi-Domain Training Versus Physical Training

Larger improvements were found after MDT than after separated physical training for verbal fluency, recall memory, and visuospatial skills (Yokoyama et al., 2015), divided attention (van het Reve and de Bruin, 2014; Yokoyama et al., 2015). Dual-task costs during walking at preferred and fast speeds were also reduced (van het Reve and de Bruin, 2014). On the other hand, neither an improvement of TMT performance after both training interventions nor a significant difference between the MDT and separated physical training groups was observed in both studies.

##### Multi-Domain Training Versus Cognitive Training

Only one (sequential training) study compared MDT to cognitive training, but the data were analyzed in three papers, which investigated the effects on cognitive functions, neurobiological mechanisms, and predictors of effectiveness either immediately after the training period (Rahe et al., 2015a,b) or at a 1-year follow-up (Kalbe et al., 2018). The short-term superiority of combined training over separated cognitive training was not supported. Only, a difference was observed for attention at follow-up, which could not be interpreted as a direct consequence of MCT (Rahe et al., 2015b).

#### Mechanisms

The frameworks of cognitive enrichment theory and exercise-induced plasticity of brain structures and functions were mentioned in few studies (Pieramico et al., 2012; Nishiguchi et al., 2015; Rahe et al., 2015a,b; Yokoyama et al., 2015; Kalbe et al., 2018). Some of them investigated more precisely the underlying mechanisms that could allow predicting/explaining the superior effects of MCT. Moreover, neither detailed hypotheses nor explanations were provided concerning the mechanisms underlying dual-task training, (e.g., Yokoyama et al., 2015).

#### Discussion

The reviewed studies showed that MDT allowed improving performance in different cognitive domains – memory (Pieramico et al., 2012; Nishiguchi et al., 2015), executive functions (Nishiguchi et al., 2015) – as compared to no training at all (see Ansai et al., 2017; for a noticeable exception). The superiority of MCT over separated physical and cognitive training was less firmly, and only partially, established. On the one hand, the studies that compared MDT and physical training found larger effects of combined training on broader domains of cognitive functions and dual-task costs (van het Reve and de Bruin, 2014; Yokoyama et al., 2015). On the other hand, the available data did not support the hypothesis that MDT resulted in superior cognitive benefits, as compared to separated cognitive training (Rahe et al., 2015a,b; Kalbe et al., 2018). These results were rather unexpected since MDT was hypothesized to be an effective training intervention that capitalizes on the combination of the effects of physical, motor, and cognitive exercises (Voelcker-Rehage et al., 2010, 2011; Pesce, 2012; Temprado et al., 2019; Temprado, 2021; Torre et al., 2021). The weakness of the effects of MDT training on brain and cognition could reflect the too low intensity of physical exercises and/or the low complexity of motor and cognitive exercises. It seems that these factors were not manipulated carefully in the reviewed studies. However, the lack of effects of MDT observed in several studies could also result from tests used to assess cognitive functions. In support of this hypothesis, Ansai et al. (2017) tested only global cognitive functions, instead of specific ones that might be more sensitive to training (i.e., attention and executive functions), while in Pieramico et al.’s (2012) study, the nature of cognitive training (sudoku, crosswords…) was presumably inadequate to improve attention or executive functions, since it only required crystallized cognition.

## General Discussion

**FIGURE 4:**
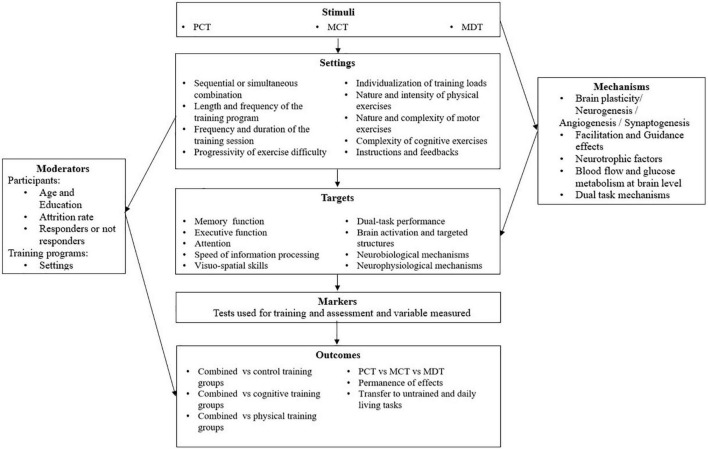
The multi-dimensional analysis of combined training filled with a brief summary of the findings of the present review.

The present work was motivated by the inconsistent conclusions provided in reviews on combined training, with regards to its superiority over separated cognitive and physical training, respectively. We attributed these inconsistencies to the lack of a concept-framed analysis. Accordingly, based on a structured background of interactive constructs, we re-analyzed available studies, with the aim to determine whether and under which conditions the superiority of conventional combined training interventions was observed (or not). The conclusions of our analysis are summarized in the Figure 4.

Concerning the stimuli (i.e., the type of training interventions), we identified three conditions that could make study protocols potentially stronger and more reliable: (i) including a comparison of four training groups (combined training, separated physical and cognitive training, and a control group), (ii) observing an effect of all the three intervention programs on cognitive functions, and (iii) assessing the differences between the combined training and the separated training groups. Over the 24 selected studies, PCT was the most represented intervention (11 studies), followed by the MCT (9) and, finally, the MDT (8). In total, 14 studies assessed the effects of combined training relative to a control group but only 8 (7 PCT and 1 MCT) compared four groups (Fabre et al., 2002; Oswald et al., 2006; Legault et al., 2011; Shatil, 2013; Linde and Alfermann, 2014; McDaniel et al., 2014; Shah et al., 2014; Desjardins-Crepeau et al., 2016). Notably, no MDT study compared four groups, suggesting that the superiority of MDT over separated training was not an important issue. This is striking since, because it combines physical, motor, and cognitive exercises, MDT was expected to be the most effective intervention, not only relative to separated training but also, and more importantly, relative to PCT and MCT. Thus, this hypothesis remains to be confirmed in further studies. Several studies only compared combined training to either cognitive or physical training alone, which did not allow to estimate the weight of each training component (i.e., physical and cognitive) in the effects of their combination. Moreover, until now, PCT, MCT, and MDT were never compared in the literature, which could be an objective for further studies (summary of the different physical/motor and cognitive stimuli of selected combined training studies in Supplementary Table 3). Concerning the settings, the average training duration was 60 min for all the three types of intervention, while the average training frequency varied from a mean of 2–3 sessions/week for the PCT and MDT to a frequency of 1 up to 3/week for the MCT. However, the large differences in training duration and frequency existing among the studies – from 7 weeks (Rahe et al., 2015a) up to 6 months (Pieramico et al., 2012) or a 1-year (Oswald et al., 2006) – and, therefore, in the total number of sessions, which precluded to identify optimal settings. Studies implementing sequential training were more numerous for PCT, while the inverse was observed for the two other types of training. Finally, there were slightly more studies using simultaneous training than sequential training. Within the different sequential protocols, cognitive training was either carried out before (i.e., Legault et al., 2011) or after (i.e. Shatil, 2013) physical training. Moreover, cognitive and physical training were sometimes carried out during separated days (e.g., Fabre et al., 2002; McDaniel et al., 2014), while in other studies, they were performed within the same day (Legault et al., 2011; Shatil, 2013; Linde and Alfermann, 2014; Shah et al., 2014; Desjardins-Crepeau et al., 2016). Notably, their time lags were not specified. This is illustrative of the more general observation that the moderators of training, which are critical to evaluate the effectiveness of the training programs, were only superficially described in most studies, thereby making the protocols not reproducible and the results difficult to explain. In particular, information relative to intensity and nature of the physical exercise, the nature and level of complexity of the motor exercises, the progressivity of exercise difficulty, etc. was often neglected, though they are known to represent the basics of physical activity guidelines to ensure training benefits. This is probably the reason why, in most studies, separated physical training failed to improve cognitive performance, while it has been widely observed in studies on separated physical and motor training (Diamond and Ling, 2020). More generally, it was often impossible to estimate whether and why the physical training component was effective (or not) to improve cognitive performance, for instance since the assessment of physical fitness was not reported (e.g., Theill et al., 2013). Also, it cannot be excluded that the combination of exercises (and how they are combined) could change the optimal dose-response relationships for physical, motor, and cognitive training. This issue remains to be addressed in future studies. All these weaknesses make the literature on combined training rather rough and like the barrel of the danaids: it can be filled endlessly with new studies, without the understanding of the generalizable principles advancing significantly. Cognitive training procedures were no more detailed in the majority of studies. Strikingly, multi-domain cognitive training (MDT), which is widely considered the most effective training strategy (Cheng et al., 2012), was scarcely used (see Rahe et al., 2015a for a noticeable exception). Fortunately, however, some studies allowed to be more optimistic, suggesting that when training programs were well designed and conducted, convincing and promising results were found (e.g., Fabre et al., 2002; Oswald et al., 2006). Actually, these two studies are the most often cited in the literature to lend credence to the superiority of combined training over separated interventions.

Concerning the targets, all the different studies were interested in the effects of training on memory, executive functions, attention, and speed of information processing, thanks to the use of specific tests, quite similar for PCT, MCT, and MDT. Notably, when global cognition was tested through MMSE or MOCA, no significant effects of training were found (e.g., Pieramico et al., 2012). Overall, we observed that, in the different studies, there were no *a priori* assumptions about the type of functions that could be affected (eventually and differentially) by each type of combined training. Finally, only a few studies investigated training-induced changes in brain structures and functions (Pieramico et al., 2012), or physiological and neurobiological underpinnings of the observed effects (Shah et al., 2014; Rahe et al., 2015a; Kalbe et al., 2018). Consequently, the mechanisms underlying the effectiveness of the different types of combined training, in particular relative to separated training, remain unclear (see below). We also observed that none study included separate analyses of responders and non-responders to training interventions, which might allow us to refine the results observed for the whole groups of participants (see Temprado et al., 2019 for an illustrative example).

With respect to the outcomes, the comparisons with control groups showed that PCT, MCT and MDT were all effective to improve cognitive performance. Attention, memory, information processing speed, and executive functions were seemingly the most sensitive domains to the effects of combined training, for both sequential and simultaneous training and the three training types. It has been hypothesized elsewhere that simultaneous training could be more effective due to the intrinsic link between cognition and physical effort (Tait et al., 2017; Herold et al., 2018), but none of the reviewed studies allowed to confirm this hypothesis. Notably, the advantage of simultaneous training could be hidden in some cases since sequential training resulted in a longer training time. Further studies are thus needed to address this issue. Only a few studies investigated the permanence of the effects of training (through follow-up) (Oswald et al., 2006; Linde and Alfermann, 2014; Eggenberger et al., 2015a,b; Kalbe et al., 2018) and the transfer to untrained tasks (McDaniel et al., 2014). Thus, findings were insufficient to draw firm conclusions, in particular concerning the permanence of training effects, due to the lack of control of (physical) activities of participants in between the different assessment sessions of the follow-up. Whether transfer of training effects eventually differed between PCT, MCT, and MDT were impossible to estimate. Also, the question remains of whether combined training led to larger transfer than separated, cognitive and physical, training.

In principle, depending on whether complex motor skills and/or aerobic training were associated with cognitive training, different processes could be impacted by PCT, MCT, and MDT. Based on the present review, it remains, however, unclear whether it was indeed the case, since most studies did not test *a priori* assumptions and, rather, were fishing for significant effects by multiplying the number of measurements. Also, it is still unknown whether differences might exist between PCT, MCT, and MDT in their effectiveness to improve performance in the different cognitive domains, since no study addressed this issue until now.

The positive effects of combined training, relative to inactivity (i.e., control groups), were well established, though not surprising. Less expected were the results of the comparison with separated physical and cognitive training. Concerning physical training, only a few studies reported both a positive effect of separated physical training on cognition and larger benefits of combined training over physical training (Fabre et al., 2002; Eggenberger et al., 2015a; León et al., 2015; Desjardins-Crepeau et al., 2016). Thus, the superiority of combined training over separated physical training was qualitatively confirmed, but only weakly quantitatively established. A possible reason is that physical training programs were ill-designed in most studies (e.g., to low intensity, no individualization, no progressive increase in intensity or difficulty…). Anyway, when observed, differences between combined training and physical training could be attributed to the positive role of additional cognitive training, which magnified the effects of physical training on cognitive performance. The question remains, however, of whether combined training could be more effective than cognitive training alone when physical training fails to improve cognitive performance. In other words, it might be speculated that even low intensity of physical activity could magnify the effects of additional cognitive exercises during combined training interventions. In this respect, the superiority of combined training over cognitive training alone was less frequently observed in the reviewed studies. This might result from the higher effectiveness of cognitive training to improve cognitive performance, relative to physical training alone. Anyway, when cognitive training was effective to improve cognitive performance, while the physical training group was not (or only weakly) effective, the effects of combined training could be mainly attributed to the weight of the load incurred by cognitive exercises. Moreover, on the basis of theoretical models of the expected effects that is, either the “facilitation + guidance hypothesis” or the Adaptive Capacity Model (Raichlen and Alexander, 2017), one should expect to observe, at least, an additive effect (i.e., 1 + 1 = 2) or at best an over-additive effect (e.g., more or less large, 1 + 1 = 5 or even, 10). Unfortunately, evidence is lacking in the reviewed studies to document these hypotheses. Unfortunately, the lack of well-designed studies precluded investigating the over-/under-additive effects of combining cognitive and physical/motor training, relative to their separated effects. This analysis could be carried out in only one study (Fabre et al., 2002), suggesting an under-additive effect of the combination. It remains, however, to be confirmed. Moreover, whether PCT, MCT, and MDT differed in their effectiveness, relative to separated training, cannot be established since none of the reviewed studies compared the different training interventions. Once again, further works are needed to address this issue, in particular, to test the potential superiority of MDT over the other training interventions. The hypothesis that MDT should be the most effective training intervention remains, however, to be confirmed thanks to conceptually-grounded training protocols (e.g., Torre et al., 2021).

How cognitive exercises must be implemented, in conjunction with physical and/or motor exercises, through simultaneous training to be more effective is also an important issue, which was not addressed in the reviewed studies. In this respect, Herold et al. (2018) distinguished two types of combination (i.e., Thinking while Moving and Moving while Thinking), which essentially differ in the way cognition is assembled (i.e., simply added or embedded) in motor tasks (see also Temprado, 2021; Torre et al., 2021 for illustrative examples). However, all the reviewed studies on simultaneous training used classic dual-task training situations (i.e., Thinking while moving), so that the comparison of the two types of training situations was impossible and could be an objective for future works (see Herold et al., 2018; Temprado, 2021 for a theoretical development; Torre et al., 2021, for a study protocol). A related question, in this respect, is whether natural activities that associate multi-domain stimulations (e.g., Tai Chi, Dance, or Nordic Walking) could be more effective than lab-customized MDT interventions to improve brain and cognition. This hypothesis is supported by the findings reported by Diamond and Ling (2020) in a recent review. It is also consistent with the results of a previous study in which Nordic Walking has been demonstrated to be highly effective to improve cognition, a least equally to MDT implemented through circuit training (Temprado et al., 2019). This could be attributed to the “moving while thinking” nature of Nordic walking tasks (see Temprado et al., 2019, for detailed discussions).

References to the mechanisms underlying the effects of training were superficial in the majority of the reviewed studies. Brain plasticity (i.e., neurogenesis, angiogenesis, and synaptogenesis), presumably stimulated by the release of neurotrophic factors (BDNF and IGF-1) resulting from physical/motor activities, was currently considered the structural and functional supports of cognitive benefits. Increase in blood flow and glucose metabolism was also frequently mentioned. On the other hand, the mechanisms at work during either cognitive or complex motor skills training were scarcely considered. More generally, whether specific mechanisms (and which ones?) could be involved in combined training, relative to separated training, was seemingly not considered an important issue. At best, the complementarity of facilitation and guidance effects was mentioned but whether guidance effects might differ depending on the type of motor-cognitive association (i.e., in dual-task or Moving while Thinking situations) was never addressed in the reviewed studies.

## Limitations of the Present Work

A first limitation of the present work is that our analysis only included the effects of combined training on brain and cognition. In future reviews, biomechanical (e.g., muscular), behavioral, physical, and motor domains should also be analyzed, thanks to the same framework. Additional studies should be considered in this respect, which does not necessarily include cognitive assessments, since several studies on conventional combined training were interested either in behavioral or cognitive outcomes, not necessarily in both or in their relation. Another limitation is that we did not analyze the training studies investigating the practice of Tai Chi, dance, or Nordic walking, which can be considered as natural forms of MDT. This was a deliberated choice since few of these studies, if any, have compared the effects of natural multi-domain activities with those of conventional combined training interventions, in particular MDT. This issue could be systematically addressed in future studies.

## Conclusion and Perspectives

Finally, we do agree with the conclusions made in previous reviews that the available studies on combined training were extremely heterogeneous. Consequently, even after a careful analysis, it was difficult to identify general principles underlying the effectiveness of combined training. At least, it can be assumed that, when they are well-designed and well-conducted (which was only scarcely the case), PCT, MCT, and MDT have the potential to be more effective than separated physical and cognitive training to improve brain and cognition.

Our review also helped to identify some still unanswered questions, which could be addressed in future studies. First of all, one can suggest that these studies should, at least, systematically compare four groups, while creating the conditions for physical and cognitive training alone to produce observable benefits for brain and cognition. Particular attention should be paid to MDT, which is predicted to be the most effective intervention. Also, sequential and simultaneous training could be tested separately and then compared to each other. Simultaneous MDT training studies should include a systematic comparison of Thinking while Moving and Moving while Thinking training situations (see Torre et al., 2021 for an illustrative example). Ideally, future studies should all use quite similar protocols in terms of duration, frequency, intensity, etc. However, how a consensus on the gold standard settings could be reached within the research community interested in exercise and cognition remains to be determined. We are making suggestions in this respect (see Supplementary Table 4). Last but not least, particular attention should be paid to the moderators that make some participants responsive and others, not responsive to the different forms of (combined) training. Finally, the next step for future work could be the application of our framework to reviewing the literature on exergames, which are currently viewed as more promising training solutions than conventional ones (Stojan and Voelcker-Rehage, 2019; Temprado, 2021).

## Author Contributions

MT and J-JT selected the reviews, meta-analyzes, the studies under consideration, and revised the initial version. They elaborated the structured framework together and they both analyzed the studies according to the model. MT wrote the first draft of the manuscript. J-JT contributed to critically revising the initial version. Both agreed with the final approval of the version to be published and agreed to be accountable for all aspects of the work.

## Conflict of Interest

The authors declare that the research was conducted in the absence of any commercial or financial relationships that could be construed as a potential conflict of interest.

## Publisher’s Note

All claims expressed in this article are solely those of the authors and do not necessarily represent those of their affiliated organizations, or those of the publisher, the editors and the reviewers. Any product that may be evaluated in this article, or claim that may be made by its manufacturer, is not guaranteed or endorsed by the publisher.
