# Supplementary material for: A Review of Combined Training Studies in Older Adults According to a New Categorization of Conventional Interventions
Source: Front Aging Neurosci. 2022 Feb 1;13:808539. doi: 10.3389/fnagi.2021.808539 (PMC8844451; doi:10.3389/fnagi.2021.808539)
Supplement: Supplementary file 1 [file Table_1.docx]

# Supplementary Material

Table 1. Definitions, effects and underlying mechanisms of separated physical, motor and cognitive training.

| **Physical training**  Includes endurance (aerobic) and muscular resistance training | | **Motor training**  Refers to the extensive practice of complex motor skills | **Cognitive training**  Refers to the extensive practice of mental task that require a one or several cognitive processes to be solved |
| --- | --- | --- | --- |
| *Definitions*  **Endurance training** targets the cardiovascular system thanks to whole-body activities (walking, running, cycling, rowing…) that increase energy expenditure to reach moderate intensity level (i.e., 60-70 % of Vo2 max) during prolonged exercise (i.e., > 30 min). Thus, endurance training enhances aerobic capacity, which allow maintaining performance during long-duration exercises (walking, cycling...) thereby developing the so-called cardiorespiratory fitness (CRF). | *Definitions*  **Muscular resistance training** is characterized by body movements aiming at increasing neuromuscular control and muscle force, which are strongly altered by aging, and correlated with decline in brain health and cognitive performance (Chen et al., 2015; Frith et al., 2018; Nakamoto et al., 2012). Basically, muscular resistance training consists of performing repeated movements with loads (expressed as % of the maximum possible load on a single repetition). To develop strength endurance, we usually use sets of 20 to 25 repetitions with loads equal to 40-60% of the maximum. Pure strength can be developed by increasing the load (80% of the maximum) which leads to a reduction in the number of repetitions (3 to 5). | *Definitions*  **Motor training** results from the practice of complex movements that is, movements that require the coordination of several degrees of freedom (multi-joint, multi limb…) and involve attentional and executive processes to be elaborated and accurately controlled (Voelcker-Rehage et al., 2010, 2011). Thus, complex motor skill training offers a possible bridge between cognitive and motor exercises (Moreau et al., 2015; Pesce 2012; Sleimen-Malkoun et al., 2013; Temprado et al., 2020; Voelcker-Rehage et al., 2010). | *Definitions*  **Cognitive training** improves brain functioning and cognitive performance through the either stimulation of specific cognitive resources or multi-tasking (Hultsch et al., 1999; Hertzog et al., 2008; Gates & Valenzuela, 2010; Stine-Morrow et al., 2014). It can be delivered thanks to either classic paper and pencil supports or through digital supports presented either on a computer or a tablet. |
| *Effects*  Endurance training has positive effects on brain plasticity and cognition. For instance, older adults having a higher level of CRF are known to be more protected against general cognitive decline than less fit elderly people (Barnes et al., 2003). Moreover, it has been shown that selective attention, episodic memory, processing speed, and executive functions are very sensitive to endurance training (Netz 2019; Diamond and Ling 2016, 2019).  *Mechanisms* | *Effects*  Resistance training has been shown to be appropriate to improve cognitive functioning, in particular executive functions and information processing speed.(Cassilhas et al., 2007 ; Herold et al., 2019 ; Wilke et al., 2019 ; Landrigan et al., 2019).  *Mechanisms* | *Effects*  It has been shown that complex motor skills training allows improving information processing speed, visuospatial capacities, and executive functions, differently however from physical (aerobic) training (Voelcker-Rehage et al., 2011). Also, it enhances the association of high neuromuscular demands and cognitive processes to finely control movement organization and execution in older adults (e.g., balance control, grasping control, interlimb coordination, adapting gait to obstacles, aiming static or moving targets).  *Mechanisms* | *Effects*  Positive training effects were observed in several studies (e.g., Green and Bavelier, 2003, 2006; Anguera et al., 2013), but inconsistent results were also reported (for meta-analyses, see Lampit et al., 2014; Simons et al., 2016; Toril et al., 2014). Cognitive training has been shown to be limited to the targeted capacities. However,it is also frequently assumed that both multi-domain cognitive training (Basak et al., 2020; Roheger et al., 2021) andstrategy-based cognitive training (e.g., Chapman et al., 2015) may induce more generalized effects and more maintenance in healthy older adults than those observed after training specific processes.  *Mechanisms* |
| With respect to neuroplasticity, a critical role seems to be played by neurotrophic factors released at blood level as a result of aerobic physical exercise (e.g., Brain-Derived Neurotrophic Factors – BDNF -- and Insulin-like Growth Factor-1 -- IGF-1 -- production). Presumably, neurotrophic factors mediate structural and functional changes observed in the brain and, finally, improvement of cognitive functioning. | Brain-Derived Neurotrophic Factors (BDNF) and Insulin-like Growth Factor-1 (IGF-1) production that result from muscular resistance training (e.g., Cotman et al., 2002; Cassilhas et al. 2007) seem also play a positive role in neuroplasticity and enhancement of cognitive performance. | The neurobiological pathways by which motor skill training affects cognition are still unclear. However, Grégorie et al. (2019) reported that motor skill training increases BDNF levels, more than combined resistance and aerobic training. Thus, motor skill training might lead to larger benefits on cognition than other types of physical training that is, endurance, muscular resistance and their combination (Netz, 2019). | At least, it can be hypothesized that cognitive training improves relevant processes in one task situation. The improvement may generalize to another task situation if there is a process overlap between situations (Taatgen, 2013). In this respect, multi-domain or strategy-based training may facilitate this overlap and, consequently, the transfer of training.  With respect to computerized cognitive training, recent studieshave identified the effective specifications of cognitively demanding digital environments, which make video games effective to stimulate brain and cognition (for details, see Bediou et al., 2018; Dale et al., 2020).  Furthermore, few studies found that distinct benefits can be realized from either cognitive (strategy-based) and physical training. Namely, the cognitive training improved executive function whereas physical training enhanced memory.At brain level, these different impacts were associated with distinct neurovascular mechanisms and networks’ connectivity (e.g., for details, see Chapman et al., 2016, 2017). |

Table 2. Included reviews and studies. Studies are classified by type of combined intervention: PCT = physical-cognitive training; MCT= motor-cognitive training; MDT= multi-domain training.

| **Reviews** | **PCT** | **MCT** | **MDT** |
| --- | --- | --- | --- |
| Law et al., 2014 | **Sequential** | | |
| Wollesen&Voelcker– Rehage, 2013 | Fabre et al., 2002 | Oswald et al., 2006 | Pieramico et al., 2012 |
| Zhu et al., 2016 | Legault et al., 2011 |  | Van het Reve & de Bruin, 2014 |
| Laurenroth et al., 2016 | Shatil, 2013 |  | Rahe et al., 2015a |
| Levin et al., 2017 | Linde & Alfermann, 2014 |  | Rahe et al., 2015b |
| Tait et al., 2017 | Shah et al., 2014 |  | Kalbe et al., 2018 |
| Gheysen et al., 2018 | McDaniel et al., 2014 |  |  |
| Joubert &Chainay, 2018 | Desjardins- Crépeau et al., 2016 |  |  |
| Gavelin et al., 2020 | **Simultaneous** | | |
| Wollesen et al., 2020 | Theill et al., 2013 | Hiyamizu et al., 2011 | Ansai et al., 2017 |
| Gallou et al., 2020 | Leon et al., 2015 | Marmeleira et al., 2009 | Yokoyama et al., 2015 |
| Guo et al., 2020 | Nourouzi et al., 2019 | Falbo et al., 2016 | Nishiguchi et al., 2015 |
|  | Eggenberger et al., 2015a |  | Jardim et al., 2021 |
|  | Eggenberger et al., 2015b |  |  |

Table 3. Summary of the different physical, motor and cognitive exercises proposed in selected combined training studies (PCT, MCT and MDT). Sequential and simultaneous protocols are distinguished.

|  | **Physical-Cognitive Training (PCT)**  Endurance effort and/or muscular resistance exercises + cognitive training (conventional or computerized) | | **Motor-Cognitive Training**  Complex motor skills + cognitive training (conventional or computerized) | | **Multi-domain Training**  Endurance effort and/or muscular resistance exercises + complex motor skills + cognitive training (conventional or computerized) | |
| --- | --- | --- | --- | --- | --- | --- |
| **Sequential** | Walking/running (Desjardin-Crepeau et al., 2016; Fabre et al., 2002; Legault et al., 2011; Linde & Alfermann 2014; McDaniel et al., 2014; Shah et al., 2014).  Cycling (Legault et al., 2011)  Full-body movements (Shatil, 2013) | Paper and pencil cognitive training (Fabre et al., 2002; Linde & Alfermann 2014)  Computerized cognitive tasks (Legault et al., 2011) or brain games (Shatil, 2013; Shah et al., 2014) | Coordination, balance, flexibility, agility (Oswald et al., 2006) | Paper pencil cognitive training (Oswald et al., 2006) | Aerobic exercise (e.g., walking) and muscular resistance training, together with the practice of complex motor skills (e.g., balance control, dancing, throwing balls to targets, stepping tasks) (Pieramico et al., 2012; Van Het Reve & De Bruin, 2014; Rahe et al., 2015a; Rahe et al., 2015b; Kalbe et al., 2018) | Paper and pencil cognitive training (e.g., crossword, sudoku, puzzle; Pieramico et al., 2012)  Computerized cognitive tasks (Van Het Reve & De Bruin 2014; Rahe, et al., 2015a) |
| **Simultaneous** | Walking (Eggenberger et al., 2015a; Theill et al., 2013)  Muscularresistance (Leon et al., 2015 ; Nourouzi et al., 2019). | Dual-task cognitive exercises | Coordination, balance control, psychomotor reactivity, flexibility, agility (Marmeleira et al., 2009 ;Hiymizu et al., 2011 ; Falbo et al., 2016) | Dual- task cognitive exercises | Aerobic exercise (e.g., walking) and muscular resistance training, together with the practice of complex motor skills (e.g., balance control, dancing, throwing balls to targets, stepping tasks) (Ansai et al., 2017; Yokoyama et al., 2015; Nishiguchi et al., 2015; Jardim et al., 2021) | Dual-task cognitive exercises |

Table 4. Proposed Gold Standards (GS), identified on the basis of the different constructs of our framework, to be considered in future studies to build effective combined training programs (PCT, MCT and MDT) and contributive studies relative to the available literature. Gold standards are meaningful since experimenters are tempted recommending doing everything possible at best. Accordingly, ideal design features proposed below as Gold Standards were ranked either as: Necessary (N), Highly Recommended (HR), Recommended (R) or Optional (O). N = conditions necessary to ensure the effectiveness of the training. HR = conditions strongly recommended to ensure the quality of the study. R = conditions recommended to increase the interest of the study. O = optional conditions to increase the quality and interest of the study. However, more “realistic” recommendations, with respect to feasibility, i.e., a kind of Minimum Viable Product (MVP), could also be helpful. In this respect, by default, the studies by Fabre et al. (2002) and Oswald et al. (2006) can be considered as MVP. On the other hand, no available study has been found to be considered as possible MVP for MDT.

| **Stimuli** | **Physical Cognitive Training**  MVP = Fabre et al.(2002) | **Motor Cognitive Training**  MVP = Oswald et al.(2006) | **Multidomain Training**  No available MVP |
| --- | --- | --- | --- |
|  | - Including a comparison of 4 groups (passive control, physical, motor and combined PCT, MCT or MDT). (HR) - Including a comparison with natural motor activity (Tai Chi, Dance, Nordic Walking) (Temprado et al., 2019) (R). - Designing separate training programs of sufficient intensity/complexity to produce effects on cognition and physical / motor abilities (HR). - Assessing systematically the differences between the different training programs (N).   **Multidomain training is expected to be the more effective training solution** | | |
| **Setting**  **Settings** | - Simultaneous combination (HR) - Frequency (2/3Xweek) (HR). - Total number of sessions (> 24) (HR) - Supervised training by experienced and specialized coaches (N). - Individualizing exercise difficulty and complexity (N). - Increasing progressively difficulty and complexity (N). - Providing frequent individualized feedbacks (N). - Testing “Moving while Thinking” training situations (Herold et al., 2018; Torre et al., 2021) (O). - Using computerized cognitive training (O). - Using Multi-domain cognitive training (R). | | |
|  | - Duration of session (45/60 min/session) (HR). - Intensity of aerobic effort (60-80% of Vo2max) (HR). | - Duration of session (45/60 min/session) (HR). - Complexity of motor skills: including a large number of degrees of freedom (joints, limbs), requiring control of speed-accuracy trade-off, taxing balance control, perturbing perception (proprioception, vision) (N). | - Duration of session (45/60 min/session) (HR). - Intensity of aerobic effort (60-80% of Vo2max) (N). - Complexity of motor skills: including a large number of degrees of freedom (joints, limbs), requiring control of speed-accuracy trade-off, taxing balance control, perturbing perception (proprioception, vision) (N). |
| **Target** | - Targeting at least EF (HR), attention (HR), information processing speed (R) and memory (R) and other functions (O) using classic laboratory tests - Targeting dual-task performance (O). - Targeting physical capacities (muscular force, muscular resistance, endurance capacities) (N). - Targeting motor capacities (balance, coordination, mobility, agility, psychomotor reaction time) (N). | | |
| **Markers** | - Using different tests for each cognitive function (R). - Using complementary laboratory and field tests to assess physical and motor capacities (R). - Testing permanence of effects and transfer (R). | | |
| **Moderators** | - Age(O). - Gender (O). - Education (N). - Baseline performance level (HR). - Motivation (O). - Compliance (intention to treat). (R) - Distinction high/low adherers (HR). - Distinction responders/not responders (HR). | | |
| **Outcomes (expected)** | - Significant effects of separated training programs (physical, motor and cognitive) on cognitive performance (HR). - Significant effects of separated physical and motor training on physical and motor outcomes (HR). - Larger effects of combined training over separated training (additive or sur-additive) (O). - Permanence and transfer of training (R). | | |

Barnes, D. E., Yaffe, K., Satariano, W. A., & Tager, I. B. (2003). A longitudinal study of cardiorespiratory fitness and cognitive function in healthy older adults. *Journal of the American Geriatrics Society*, 51(4), 459-465. doi:10.1001/jamainternmed.2013.189

Basak, C., Qin, S., and O’Connell, M. A. (2020). Differential effects of cognitive training modules in healthy aging and mild cognitive impairment: a comprehensive meta-analysis of randomized controlled trials. Psychology and Aging, 35, 220–249. doi: 10.1037/pag0000442.

Cassilhas, R., Viana, V., Grassmann, V., Santos, R., Santos, R., Tufik, S., & Mello, M. (2007). The impact of resistance exercise on the cognitive function of the elderly. *Medicine & Science in Sports & Exercise*, *39*(8), 1401-1407. <https://doi.org/10.1249/mss.0b013e318060111f>

Chapman, S. B., Aslan, S., Spence, J. S., Hart, J. J. Jr., Bartz, E. K., Didehbani, N., et al. (2015). Neural mechanisms of brain plasticity with complex cognitive training in healthy seniors. *Cerebral Cortex*, 25, doi:10/1093/cercor/bht224.

Chapman, S.B., Aslan, S., Spence, J.S., Keebler, M.W., DeFina, L.F., Didehbani, N., Perez, A.M., Lu, H., D’Esposito, M. (2016). Distinct brain and behavioral benefits from cognitive vs physical training: a randomized trial in aging adults. *Frontiers in Human Neuroscience*, 10:338. doi: 103389/fnhum2016.00338

Chapman, S.B., Aslan, S., Spence, J.S., Keebler, M.W. (2017). Enhancing innovation and underlying neural mechanisms via cognitive training in healthy older adults. *Frontiers in Aging Neuroscience*, 9:314. doi:103389/fnagi.2017.00314

Chen, W. L., Peng, T. C., Sun, Y. S., Yang, H. F., Liaw, F. Y., Wu, L. W., ... & Kao, T. W. (2015). Examining the association between quadriceps strength and cognitive performance in the elderly. *Medicine*, 94(32). https://doi.org/10.1097/MD.0000000000001335

Cotman, C., &Engesser-Cesar, C. (2002). Exercise enhances and protects brain function. *Exercise and Sport Sciences Reviews*, *30*(2), 75-79. <https://doi.org/10.1097/00003677-200204000-00006>

Diamond, A., & Ling, D. (2019). Aerobic-exercise and resistance-training interventions have been among the least effective ways to improve executive functions of any method tried thus far. *Developmental Cognitive Neuroscience*, *37*, 100572. <https://doi.org/10.1016/j.dcn.2018.05.001>

Frith, E., &Loprinzi, P. D. (2018). The association between lower extremity muscular strength and cognitive function in a national sample of older adults. *Journal of lifestyle medicine*, 8(2), 99. https://doi.org/[10.15280/jlm.2018.8.2.99](https://dx.doi.org/10.15280%2Fjlm.2018.8.2.99)

Gates, N., & Valenzuela, M. (2010). Cognitive exercise and its role in cognitive function in older adults. *Current psychiatry reports*, 12(1), 20-27. https://doi.org/[10.1007/s11920-009-0085-y](https://doi.org/10.1007/s11920-009-0085-y)

Grégoire, C. A., Berryman, N., St-Onge, F., Vu, T. T. M., Bosquet, L., Arbour, N., &Bherer, L. (2019). Gross motor skills training leads to increased brain-derived neurotrophic factor levels in healthy older adults: A pilot study. *Frontiers in physiology*, 10, 410. <https://doi.org/10.3389/fphys.2019.00410>

Herold, F., Törpel, A., Schega, L., & Müller, N. (2019). Functional and/or structural brain changes in response to resistance exercises and resistance training lead to cognitive improvements – a systematic review. *European Review of Aging and Physical Activity*, *16*(1). <https://doi.org/10.1186/s11556-019-0217-2>

Hertzog, C., Kramer, A. F., Wilson, R. S., &Lindenberger, U. (2008). Enrichment effects on adult cognitive development: can the functional capacity of older adults be preserved and enhanced?. *Psychological science in the public interest*, 9(1), 1-65. [https://doi.org/10.1111/j.1539-6053.2009.01034.x](https://doi.org/10.1111%2Fj.1539-6053.2009.01034.x)

Hultsch, D. F., Hertzog, C., Small, B. J., & Dixon, R. A. (1999). Use it or lose it: engaged lifestyle as a buffer of cognitive decline in aging?. *Psychology and aging*, 14(2), 245. <https://doi.apa.org/doi/10.1037/0882-7974.14.2.245>

Lampit, A., Hallock, H., & Valenzuela, M. (2014). Computerized cognitive training in cognitively healthy older adults: a systematic review and meta-analysis of effect modifiers. *PLoS Med*, 11(11), e1001756. https://doi.org/10.1371/journal.pmed.1001756

Landrigan, J. F., Bell, T., Crowe, M., Clay, O. J., & Mirman, D. (2019). Lifting cognition: a meta-analysis of effects of resistance exercise on cognition. *Psychological Research*, 1-17. <https://doi.org/10.1007/s00426-019-01145-x>

Nakamoto, H., Yoshitake, Y., Takai, Y., Kanehisa, H., Kitamura, T., Kawanishi, M., & Mori, S. (2012). Knee extensor strength is associated with Mini-Mental State Examination scores in elderly men. *European journal of applied physiology*, 112(5), 1945-1953. <https://doi.org/10.1007/s00421-011-2176-9>

Netz, Y. (2019). Is there a preferred mode of exercise for cognition enhancement in older age?- a narrative review. *Frontiers in Medicine*, *6*. <https://doi.org/10.3389/fmed.2019.00057>

Roheger, M., Liebermann-Jordanidis, H., Krohm, F., Adams, A., Kalbe, E. (2021). Prognostic factors and models for changes in cognitive performance after multi-domain cognitive training in healthy older adults: a systematic review. Frontiers in Human Neuroscience, 15:636355. doi: 10.3389/fnhum.2021.636355.

Simons, D. J., Boot, W. R., Charness, N., Gathercole, S. E., Chabris, C. F., Hambrick, D. Z., & Stine-Morrow, E. A. (2016). Do “brain-training” programs work?. *Psychological Science in the Public Interest*, 17(3), 103-186. <https://doi.org/10.1177/1529100616661983>

Sleimen-Malkoun, R., Temprado, J.-J., Berton, E. (2013). Age-related changes of movement patterns in discrete Fitts’ task. *BMC Neuroscience*, 14, 145. <https://doi.org/10.1186/1471-2202-14-145>

Stine-Morrow, E. A., Payne, B. R., Roberts, B. W., Kramer, A. F., Morrow, D. G., Payne, L., ... &Parisi, J. M. (2014). Training versus engagement as paths to cognitive enrichment with aging. *Psychology and Aging*, 29(4), 891. [https://doi.org/10.1037/a0038244](https://psycnet.apa.org/doi/10.1037/a0038244)

Taatgen, N. A. (2013). The nature and transfer of cognitive skills. *Psychological review*, 120(3), 439. [https://doi.org/10.1037/a0033138](https://psycnet.apa.org/doi/10.1037/a0033138)

Toril, P., Reales, J. M., & Ballesteros, S. (2014). Video game training enhances cognition of older adults: a meta-analytic study. *Psychology and aging*, 29(3), 706. <http://dx.doi.org/10.1037/a0037507>

Wilke, J., Giesche, F., Klier, K., Vogt, L., Herrmann, E., &Banzer, W. (2019). Acute Effects of Resistance Exercise on Cognitive Function in Healthy Adults: a systematic review with multilevel meta-analysis. *Sports Medicine*, *49*(6), 905-916. <https://doi.org/10.1007/s40279-019-01085-x>
